# Interrater and test-retest reliability and validity of the Norwegian version of the BESTest and mini-BESTest in people with increased risk of falling

**DOI:** 10.1186/s12877-017-0480-x

**Published:** 2017-04-20

**Authors:** Charlotta Hamre, Pernille Botolfsen, Gro Gujord Tangen, Jorunn L. Helbostad

**Affiliations:** 1 Department of Physiotherapy and Department of Geriatric Medicine, Oslo University Hospital Ulleval, PB 4956 Nydalen, 0424 Oslo, Norway; 2 Department of Health Sciences, University of Oslo, Oslo, Norway; 3 Department of Physiotherapy, Oslo and Akershus University College of Applied Sciences, Oslo, Norway; 4 Norwegian National Advisory Unit on Aging and Health, Vestfold Hospital Trust and Department of Geriatric Medicine, Oslo University Hospital Ulleval, Oslo, Norway; 5 Department of Neuroscience, Norwegian University of Science and Technology, Trondheim, Norway; 6 Department of Clinical Services, St Olavs Hospital of Trondheim, Trondheim, Norway

**Keywords:** Balance, Assessment, Reliability, Validity

## Abstract

**Background:**

The Balance Evaluation Systems Test (BESTest) was developed to assess underlying systems for balance control in order to be able to individually tailor rehabilitation interventions to people with balance disorders. A short form, the Mini-BESTest, was developed as a screening test. The study aimed to assess interrater and test-retest reliability of the Norwegian version of the BESTest and the Mini-BESTest in community-dwelling people with increased risk of falling and to assess concurrent validity with the Fall Efficacy Scale-International (FES-I), and it was an observational study with a cross-sectional design.

**Methods:**

Forty-two persons with increased risk of falling (elderly over 65 years of age, persons with a history of stroke or Multiple Sclerosis) were assessed twice by two raters. Relative reliability was analysed with Intraclass Correlation Coefficient (ICC), and absolute reliability with standard error of measurement (SEM) and smallest detectable change (SDC). Concurrent validity was assessed against the FES-I using Spearman’s rho.

**Results:**

The BESTest showed very good interrater reliability (ICC = 0.98, SEM = 1.79, SDC_95_ = 5.0) and test-retest reliability (rater A/rater B = ICC = 0.89/0.89, SEM = 3.9/4.3, SDC_95_ = 10.8/11.8). The Mini-BESTest also showed very good interrater reliability (ICC = 0.95, SEM = 1.19, SDC_95_ = 3.3) and test-retest reliability (rater A/rater B = ICC = 0.85/0.84, SEM = 1.8/1.9, SDC_95_ = 4.9/5.2). The correlations were moderate between the FES-I and both the BESTest and the Mini-BESTest (Spearman’s rho −0.51 and-0.50, *p* < 0.01).

**Conclusion:**

The BESTest and its short form, the Mini-BESTest, showed very good interrater and test-retest reliability when assessed in a heterogeneous sample of people with increased risk of falling. The concurrent validity measured against the FES-I showed moderate correlation. The results are comparable with earlier studies and indicate that the Norwegian versions can be used in daily clinic and in research.

## Background

Balance is an integral part of almost every movement in everyday life [1]. Balance problems in elderly people and in people with neurologic problems are common and often associated with increased risk of falling [2–6]. Balance problems and falls are also common causes for contact with physiotherapists**.** Clinical practice guidelines state that older people should be screened for fall risk by asking questions about falls, and that a positive screening should be followed by further fall risk assessments including balance assessment and targeted interventions [7]. Thus, there is a need for balance assessment tools that can guide decision making and evaluate treatment of balance problems [8].

Clinical balance tests are commonly used to indicate if the patient has a balance problem and can benefit from an intervention. In order to guide decision making, outcome measures should assess the cause of the problems and not only reveal that it exists [2]. Outcome measures based on a systems approach for motor control are more helpful when the purpose of the assessment is to determine the underlying causes of the balance deficit [9]. The Balance Evaluation Systems Test (BESTest) was developed to assess and to differentiate between 6 underlying balance systems contributing to balance control using a “systems model of motor control” as the theoretical framework [2]. It is divided into 6 sections; I. Biomechanical constraints, II. Stability limits/verticality, III. Anticipatory postural adjustments, IV. Postural responses, V. Sensory orientation and VI. Stability in gait. A shortened form of the BESTest, the Mini-BESTest, was developed shortly after the BESTest in order to improve feasibility for clinical use [10]. The Mini-BESTest contains items from 4 of the 6 sections from the BESTest (sections III, IV, V and VI).

The original version of the BESTest has shown to have high interrater and test-retest reliability when used in subjects with neurological disease [2]. Interrater reliability assessed with an Intraclass Correlation Coefficient (ICC) (2,1) has been reported to be 0.91 for the test as a whole, and between 0.79 to 0.96 for the different sections [2]. The BESTest has also showed high test-retest result ICC(2,1) 0.88 [11]. The Mini-BESTest has also shown high interrater and test-retest reliability (all ICC values >0.94) when tested in subjects with neurological disease [11–14]. In the present study we assessed concurrent validity through examining the correlation with the Fall Efficacy Scale-International (FES-I) which is a questionnaire measuring the degree to which a person is concerned about falling in different everyday situations [15].

Most clinical balance tests have a functional approach and can indicate if the patient has a balance problem and thereby can benefit from an intervention. The BESTest seeks to differentiate between underlying balance systems contributing to balance in order not only to indicate balance problems, but to go further and to also guide decision making in treatment. Therefore, in view of its unique properties and to be able to use it in Norwegian conditions, it is a desire to translate the BESTest and the Mini BESTest. To ensure that the original intentions of the test are preserved in the national translations, the translations should follow certain specified procedures, and a reliability and validity study of the new version should be performed [16, 17].

The purpose of the present study was to determine relative and absolute interrater and test-retest reliability and smallest detectable change (SDC) of the Norwegian translation of the BESTest and the Mini-BESTest, as well as assessing the concurrent validity with the FES-I in community-dwelling people with increased risk of falling. Usually, a good correlation when measuring concurrent validity, is assumed to be above 0.75 to be considered as a strong correlation [18]. For questionnaires, there is an understanding that a good correlation is a bit lower than for physical performance tests [18].

We hypothesized that the Norwegian versions of the BESTest and the Mini-BESTest would show comparable results as the original version; demonstrating high interrater and test-retest reliability and moderate concurrent validity.

## Methods

### Translation

The BESTest and the Mini-BESTest were translated into Norwegian following international guidelines [16]. Both tests were translated into Norwegian by three experienced physiotherapists, fluent in both English and Norwegian. These three versions were compared and discussed until agreement was reached. In addition, a senior researcher commented on the translation. A professional translator then back-translated both tests to English. Throughout the translation process we had communication with the original author (Fay Horak) who gave us permission to conduct the translation and who also approved the back-translated version. In the Norwegian versions, meters and centimetres rounded up to the closest centimetre are used instead of inches and feet.

### Design and subjects

This was an observational study with a test-retest design. Three groups of participants, elderly over 65 years of age, people with a history of stroke or Multiple Sclerosis (MS), representing different clinical conditions and fall-risk profiles were recruited to ensure heterogeneity of balance impairments. In order to be included the participants had to be able to walk 6 m without a walking aid, and to be able to meet for testing on two occasions with a two-day interval. Exclusion criteria were to be unable to understand or follow oral instructions. Eligible people were asked to participate by their treating physiotherapist from three inclusion sites: The Geriatric rehabilitation ward at Oslo University Hospital (OUS), the Department of physiotherapy at Oslo and Akershus University College of Applied Sciences (HiOA) and from the Multiple Sclerosis Centre Hakadal in the period of 01.09.11–01.06.12. Forty-two participants were included in the study.

### Qualification of raters

Two raters with extensive experience conducted the assessments, rater A had 20 years of experience both as a clinical physiotherapist and as a teacher in physiotherapy education at the university, rater B had 16 years of experience as a clinical physiotherapist.

Both raters had attended a 3-day BESTest workshop led by the developer of the tests and also watched the training videos available at the BESTests web portal [19]. Before the study, the raters had three training sessions where they were allowed to discuss how to score the test items with each other. The raters were not allowed to discuss the scoring during the study period.

### Procedures

The test sessions took place at the three inclusion sites, and the same test equipment was used at all three sites. The participants were tested with two-day interval; both test sessions were conducted in the same room and at the same time of the day. Instructions to the participants were to live their normal life, and take their medication according to their normal regime during the test period. Before the second test session, the participants were asked about any relevant changes in self-perceived balance state since the first test session. The participants performed the BESTest and the Mini-BESTest barefoot except for the tasks in section VI where they were allowed to wear flat-heeled shoes. All participants used the same shoes at both sessions.

Both the BESTest and the mini-BESTest were scored at both test sessions. Rater B administered all the tests at both sessions, while both raters scored the participants performance from the same test trials. All items in the mini-BESTest are included in the BESTest, so each task (item) was only performed once and scored according to the test criteria. The participants were allowed to rest as needed during the test sessions.

Demographic information (age, weight, height, diseases, number of medications and number of falls during the last year) was obtained by interviewing the subjects before the first test session. At the end of the first session, the FES-I was administered by rater B as a structured interview. Total time for first session was approximately 60 min. The second session was without the interviewing, and took approximately 40 min to administrate.

### Assessment tools

#### BESTest

The BESTest consists of 27 different tasks, including a total of 36 items, because some tasks include testing of both the right and the left side of the body [2]. All items are scored on a 4-point ordinal scale (0–3), with higher scores indicating better balance. Test scores are calculated for each of the 6 sections and for the summary of all test items for all sections (0–108). Section scores and total score are usually converted to percent-scores. The BESTest takes approximately 35–40 min to complete.

#### Mini-BESTest

Mini-BESTest consists of 14 tasks focusing on dynamic balance [10]. The items are scored on a 3-point ordinal scale (0–2), giving a maximum score of 32 points, with higher scores indicating better balance. The Mini-BESTest takes approximately 10–15 min to administer.

#### Falls efficacy scale - international

The FES-I is a 16-item questionnaire for assessment of fall-related self-efficacy, and related to performance of common activities in a person’s everyday life [15]. The items are rated according to "how concerned you are about the possibility of falling" using a 4-point scale (1–4) with the following responses; 1) not at all, 2) somewhat, 3) fairly, 4) very concerned, giving a total score from 16 to 64 points. Higher scores indicate more concerns for falling. The Norwegian translation of the FES-I has been established in individuals with increased risk of falling [20].

### Data analysis

All statistical analyses were performed using the IBM SPSS Statistics, version 22.0 (IBM Corp., Armonk, New York). For calculation of relative and absolute reliability the criteria for evaluation of measurements developed by the prevention of Falls Network Europa [21] and The COSMIN checklist was followed [22].

The sample size calculations were based on the formula *n* = 2×(SD/Δ)2xk [23], where the expected standard deviation (SD) was based on Horak’s study with SD = ±9.6% [2]. At the time, no study had established clinical relevance change for the BESTest; we therefore chose the least clinically difference, which is the difference in score which patients perceive as important, to be 7.0% based on other clinical outcome measures [24]. We used α = 0.05 with a power of 0.80. This gave a sample size of minimum 30 participants [23].

Intraclass Correlation Coefficient (ICC) with 95% confidence interval was used as measures of relative reliability [25, 26]. For the interrater reliability the ICC(2,1) and the ICC(3,1) were used. ICC(2,1) is based on a two-ways random absolute agreement, shows variability between raters, and the results can be generalized to other raters. ICC(3,1), using two-ways mixed, consistency, is a measure of the consistency of the scoring of each rater. Systematic errors are not included as measurement error. When ICC(2,1) and ICC(3,1) are identical or shows only minor differences, there is no systematic error e.g. learning effect, present [25].

For the test-retest reliability ICC(1,1) using a one-way random model, and ICC(3,1) was used. With ICC(1,1) all systematic and random intrasubject variability is seen as measurements error. Again, if ICC(1,1) and ICC(3,1) is identical or shows only minor differences, there is no systematic error present [25].

Measurement error is the systematic and random error of a subject’s score that is not attributed to the true changes in the construct to be measured [22]. For assessment of absolute reliability Standard Error of Measurement (SEM) and Smallest Detectable Change (SDC_95_) were used. SEM represents the standard deviation of repeated measures in one participant (SEM = SD/√2). SDC_95_ represents the smallest change that a participant must show to ensure that the observed change is real and not just a measurement error (SDC_95_ = SEMx√2 × 1.96) [22]. Since the FES-I demonstrated a skewed distribution we used the Spearman Rank Correlation to examine concurrent validity between the FES-I and rater A’s total scores of the BESTest and the Mini-BESTest. Correlation coefficients of 0.00–0.25 were interpreted as little to no correlation, 0.25–0.49 as fair, 0.50–0.75 as a moderate to good, and above 0.75 as a strong correlation [18].

The presence of floor and ceiling effects was defined as 15% or more of the participants having the lowest or the respectively the highest possible score on the BESTest and the Mini-BESTest [27].

## Results

### Descriptive statistics

A sample of 42 community-dwelling people, 28 women, and 15 men participated; elderly persons (*n* = 20), persons diagnosed with stroke (*n* = 12) and persons with MS (*n* = 10). The participants characteristics are shown in Table 1. All participants completed the study procedures as described. No unexpected events or injuries were reported. None of the participants reported any changes in balance performance from the first to the second test session.

**Table 1:** Characteristics of participants

	Total	Elderly persons	Stroke	MS
n	42	20	12	10
Men, n (%)	15 (35.7)	5 (25.0)	7 (58.3)	3 (30.0)
Age (years), mean (SD)	71.7 (14.8)	77.4 (8.8)	80.0 (6.3)	50.4 (10.7)
Body mass index (kg/m^2^), mean (SD)	25.3 (4.1)	24.6 (4.5)	25.1 (4.1)	26.8 (3.4)
Walking aid, n (%)	19 (45.2)	7 (36.8)	5 (26.3)	7 (36.8)
Falls past year, n (%)	28 (66.7)	14 (50.0)	7 (25.0)	7 (25.0)
Recurrent fallers, n (%)	17 (40.5)	6 (35.3)	5 (29.4)	6 (35.3)
FES-I, (16–64), mean (SD)	24.1 (7.6)	22.5 (6.1)	22.5 (4.4)	29.4 (10.9)

*n* number of participants, *SD* standard deviation, *MS* multiple sclerosis, *FES-I* Fall Efficacy Scale-International

The scores for the BESTest and the Mini-BESTest for both raters at both test session 1 and test session 2 are shown in Table 2. The mean total score for the BESTest for the two test sessions for both raters was 82.6 points (SD = 14.5; min-max = 31–106). The mean total score for the Mini-BESTest was 19.0 points (SD = 5.0; min-max = 1–27), and for the FES-I 24.1 (SD = 7.6; min-max = 16–55). None of the participants got the lowest or highest possible score, thus no floor or ceiling effect was observed. The correlation between the total scores of the BESTest and the Mini-BESTest was *r* = 0.95, *p* < 0.001**.**

**Table 2:** Means, standard deviations and range for BESTest (section and total scores) and Mini-BESTest for rater A and rater B, first and second measurement (*n* = 42)

	Rater A						Rater B					
	1. measurement			2. measurement			1. measurement			2. measurement		
	$\overline{x}$	SD	Range	$\overline{x}$	SD	Range	$\overline{x}$	SD	Range	$\overline{x}$	SD	Range
I. Biomechanical Constraints 0–15	11.7	2.5	5–15	12.0	2.4	6–15	12.0	2.6	5–15	12.5	2.5	5–15
II. Stability limits/Verticality 0–21	17.1	3.1	2–21	18.1	1.8	13–21	17.9	3.2	2–21	18.5	2.0	13–21
III. Anticipatory Postural Adjustments 0–18	11.9	3.2	4–18	12.9	2.6	6–18	12.0	3.1	4–18	13.1	3.0	3–18
IV. Postural Responses 0–18	11.8	3.7	4–18	12.9	2.6	6–18	12.0	3.1	4–18	13.1	3.0	3–18
V. Sensory Orientation 0–15	12.0	3.3	0–15	12.4	3.1	0–15	12.1	3.3	0–15	12.4	3.1	0–15
VI. Stability in Gait 0–21	14.9	4.0	4–21	15.6	3.3	6–20	15.3	4.2	3–20	15.6	3.4	5–20
BESTest total score 0–108	79.6	15.0	31–100	83.8	13.5	35–101	81.5	15.4	31–106	85.3	13.9	31–102
Mini-BESTest 0–28	18.3	5.2	1–27	19.3	4.7	3–26	18.6	5.4	1–27	19.7	4.7	1–26

$\overline{x}$=mean, SD = standard deviation

### Reliability

#### BESTest

Interrater reliability of the total score and the section scores of the BESTest are presented in Table 3. Relative reliability for the total score was ICC(2,1) = 0.98, and between 0.87 (section I) and 0.99 (section V) for the section scores. ICC(2,1) showed only minor differences compared with ICC(3,1) (ICC(3,1) = 0.99, and between 0.87 (section I) and 0.99 (section V), indicating that there was no systematic differences in scores between raters [21]. Absolute reliability analysed with SEM for the total score was 1.79, with a SDC_95_ of 5.0 points (Table 3).

**Table 3:** Relative and absolute interrater reliability for the BESTest (section and total scores) and Mini-BESTest, first test session

	ICC (2,1)	95% CI	ICC (3,1)	95% CI	SEM	SDC 95%
Section I	0.87	0.77–0.93	0.87	0.77–0.93	0.93	2.57
Section II	0.92	0.69–0.97	0.95	0.90–0.97	1.39	3.85
Section III	0.97	0.94–0.98	0.97	0.94–0.98	0.56	1.55
Section IV	0.96	0.92–0.98	0.96	0.93–0.98	0.71	1.98
Section V	0.99	0.99–0.99	0.99	0.99–0.99	0.22	0.60
Section VI	0.94	0.89–0.97	0.94	0.89–0.97	1.01	2.79
BESTest total	0.98	0.91–.99	0.99	0.97–0.99	1.79	4.96
Mini-BESTest total score	0.95	0.91–0.97	0.95	0.91–0.97	1.19	3.30

*ICC* intraclass correlation coefficient, *CI* confidence interval, *SEM* standard error of measurement, *SDC* smallest detectable change

The relative reliability for test-retest showed an ICC(1,1) of 0.89 for the total scores and between 0.49 (section II) and 0.86 (section VI) for the section scores (Table 4). The test-retest reliability also demonstrated small differences between ICC(1,1) and ICC(3,1) (ICC(3,1) = 0.93(rater A)/0.92(rater B), and between 0.53 (section II) and 0.87 (section V)) which suggest no learning effect between test and retest. Absolute reliability analysed with SEM for the total score was 3.9 points for rater A and 4.3 for rater B, which gives a SDC_95_ equal to 10.8 points (corresponding percent score is 10%) for smallest difference between the first and the second assessment for rater A and 11.8 (10.9% score) for rater B.

**Table 4:** Relative and absolute test-retest reliability for the BESTest (section and total scores) and Mini-BESTest, first test session

		ICC(1,1)	95% CI	ICC(3,1)	95% CI	SEM	SDC 95%
Seksjon I	A	0.68	0.48–0.82	0.68	0.48–0.82	1.39	3.85
	B	0.70	0.50–0.82	0.71	0.51–0.83	1.39	3.85
Seksjon II	A	0.49	0.22–0.69	0.53	0.27–0.72	1.72	4.78
	B	0.53	0.28–0.72	0.54	0.29–0.73	1.80	4.99
Seksjon III	A	0.78	0.63–0.88	0.83	0.71–0.91	1.18	3.28
	B	0.77	0.60–0.87	0.82	0.69–0.90	1.29	3.58
Seksjon IV	A	0.65	0.44–0.80	0.68	0.47–0.81	1.99	5.53
	B	0.73	0.55–0.85	0.75	0.58–0.86	1.76	4.88
Seksjon V	A	0.86	0.76–0.92	0.87	0.77–0.93	1.17	3.23
	B	0.85	0.55–0.92	0.85	0.75–0.92	1.23	3.40
Seksjon VI	A	0.83	0.71–0.91	0.85	0.73–0.92	1.42	3.93
	B	0.82	0.69–0.90	0.82	0.69–0.90	1.63	4.51
BESTest total score	A	0.89	0.80–0.94	0.93	0.87–0.96	3.90	10.81
	B	0.89	0.80–0.94	0.92	0.85–0.95	4.28	11.84
Mini-BESTest	A	0.85	0.74–0.92	0.87	0.77–0.93	1.78	4.94
	B	0.84	0.73–0.91	0.86	0.76–0.92	1.88	5.22

*ICC* intraclass correlation coefficient, *CI* confidence interval, *SEM* standard error of measurement, *SDC* smallest detectable change

#### Mini-BESTest

Table 3 presents the result for interrater reliability for the total score for the Mini-BESTest, with ICC(1,1) of 0.95 and SEM of 1.19 with a SDC_95_ of 3.3 points. Table 4 presents the result for test-retest reliability ICC(1,1) of 0.85 and 0.84 (rater A and rater B), SEM 1.8 and 1.9, and SDC_95_ 4.9 and 5.2, respectively.

### Validity

There were moderate correlations for the BESTest and the Mini-BESTest against the FES-I; *r*_s =_-0.51 (*p* = 0.01) and *r*_s =_-0.50 (*p* = 0.01), respectively.

## Discussion

The present study aimed to determine reliability and concurrent validity of the Norwegian version of the BESTest and its short form, the Mini-BESTest in community-dwelling people with increased risk of falling. Both versions of the test demonstrated very good reliability. SEM was 3.9–4.3 for the BESTest total score and 1.8–1.9 for the Mini-BESTest, while the SDC was 10.8–11.8 points for BESTest and 4.9–5.2 points for the Mini-BESTest. The study showed moderate correlations between the two BESTests and FES-I.

### Reliability

The absolute reliability, presented by SEM and SDC in actual scale units, is probably the most important reliability measures for clinical purposes. SDC values of 6.9 have previously been reported for the BESTest [28], and in the range of 2.0–4.4 points on the Mini-BESTest [12, 13, 28–30]. We found SDC to be 10.8–11.8 for the BESTest, and 4.9–5.2 for the Mini-BESTest, for the two raters in our study. The discrepancy between our results and the previous studies may be explained by the fact that we used two raters and did not score the tests from video as have been done in the other studies. Furthermore, we included patients with neurological disease who have earlier been found to have high variability in behaviour [31], which might also explain the larger SDC in our study. Biological variability is however a characteristic by the sample and should not be regarded a measurement error.

### Validity

Ideally, the concurrent validity of newly developed assessments methods should be established by examining how well the new method reflects the existing gold standard method. However, because the BESTest is developed based on other balance and mobility tests such as the Berg Balance scale, the Dynamic Gait Index and the Timed up and Go, and incorporates modified items from these tests, this is not a suitable approach for evaluation of the validity of the BESTest [2]. Similar to previous studies we chose to examine concurrent validity of the BESTest with measures that addresses related, but not identical constructs. While previous studies have used the ABC Scale, we used the FES-I in our study since the Norwegian translation has previously been translated to Norwegian and tested for psychometric properties [20, 32]. The correlations between the FES-I and the BESTests in our study were moderate (BESTest *r*_s_ = −0.51/Mini-BESTest *r*_s_ = 0–.50). This is in accordance with our a priori hypothesis, as fear of falling and balance are related but not identical constructs. Fear of falling is naturally related to balance performance, however other factors than balance will also influence fear of falling. Previous studies have observed moderate to high correlations between the BESTest and the ABC Scale [2, 28, 30, 33]. Although the ABC Scale and the FES-I have similar items and are highly correlated (*r* = 0.68), there are also differences between the scales. The ABC Scale has more questions on gait, while the FES-I has more focus on social activities [34]. This may also explain the higher correlations between the BESTests and the ABC Scale compared to our findings.

The study sample in a reliability and validity study should reflect the population of interest [24]. To cover a heterogeneous population of people at risk of falling we included subgroups of older persons and persons with diagnoses of stroke or MS. All three groups have earlier been found to have increased risk of falling, and are thought to display a variety of balance impairments [1, 6, 35, 36]. We succeeded to recruit a heterogeneous sample, as the total scores for the BESTest ranged from 31 to 102 and there was also a wide range of scores for the different test sections. Correspondingly, the scores for the Mini-BESTest showed a considerable variability with scores ranging between 1 and 27 points (max score 28) (Table 2). Thus, our results can likely be generalised to a wide group of people with increased risk of falling.

A major strength of the study is the thoroughly conducted test procedures. Both versions of the BESTest were translated according to cross-cultural validity procedures [16]. Further, the two test sessions were close to identical as all the participants are tested by the same raters, with the same equipment in the same room at the same time of day. Another strength of the study is the evaluation of absolute reliability of the BESTest and the Mini-BESTest. By determining SEM and SDC_95_ for all subscales and total scores in a sample of persons with fall risk, this increases the interpretability of BESTest results both in clinical practice and in research.

The study findings will be useful for directing interventions and fall-preventions aimed at reducing falls and improving balance in patients coming to the physiotherapist for treatment. Conclusively this study indicates that the Norwegian version of BESTest and its short form the Mini-BESTest are reliable and valid instruments for assessing balance in community-dwelling people with increased risk of falling, but that change in balance performance measured with the BESTest and the Mini-BESTest should be interpreted cautiously.

## Conclusion

The Norwegian version of the BESTest and the Mini-BESTest are reliable and valid instruments for assessing balance in community-dwelling people with increased risk of falling. The results are comparable with the original versions and indicate that the Norwegian versions can be used in daily clinic and in research.

## Abbreviations

BESTest: Balance evaluation systems test
FES-I: Fall Efficacy Scale – International
ICC: Intraclass correlations coefficient
SEM: Standard error of measurement
SDC: Smallest detectable change
ABC Scale: Activities-specific balance confidence scale
